# Cognitive–behavioural therapy by psychiatric trainees: can a little knowledge be a good thing?

**DOI:** 10.1192/pb.bp.113.046029

**Published:** 2015-02

**Authors:** Eric Kelleher, Melissa Hayde, Yvonne Tone, Iulia Dud, Colette Kearns, Mary McGoldrick, Michael McDonough

**Affiliations:** 1 Department of Psychiatry, Trinity College Dublin, Ireland; 2 St Patrick’s University Hospital, Dublin, Ireland; 3 St James’ University Hospital, Dublin, Ireland; 4 Student Counselling Service, Trinity College, Dublin, Ireland

## Abstract

**Aims and method** To establish the competency of psychiatric trainees in delivering cognitive–behavioural therapy (CBT) to selected cases, following introductory lectures and supervision. Supervisor reports of trainees rotating through a national psychiatric hospital over 8.5 years were reviewed along with revised Cognitive Therapy Scale (CTS-R) ratings where available. Independent *t*-test was used to compare variables.

**Results** Structured supervision reports were available for 52 of 55 (95%) trainees. The mean result (4.6, s.d. = 0.9) was at or above the accepted level for competency (≥3) for participating trainees. Available CTS-R ratings (*n* = 22) supported the supervisor report findings for those particular trainees.

**Clinical implications** This study indicates that trainees under supervision can provide meaningful clinical interventions when delivering CBT to selected cases. The costs of supervision need to be judged against these clinical gains.

Developing competencies in psychotherapy is a requirement of basic psychiatry specialist training in both the UK and Ireland.^1,2^ Of the psychotherapies, cognitive–behavioural therapy (CBT) has gained increasing prominence both as a treatment alternative and adjunct to medication due to its proven efficacy in the treatment of mood,^3^ anxiety,^4^ psychotic^5^ and eating disorders.^6^ In England, the Improving Access to Psychological Therapies (IAPT; www.iapt.nhs.uk) outlines the government’s commitment to use CBT in the future.^7^ This highlights a clear practical need for all psychiatric trainees to develop competencies in CBT.

In clinical practice, psychotherapy is often delivered by psychotherapy trainees under supervision, such as post-doctoral fellows or pre-doctoral interns in psychology or social work.^8^ Previous work by Brittlebank & Owens suggests that psychiatric trainees can deliver CBT effectively to patients.^9^ To date, much of the literature on psychiatric trainees delivering CBT has focused on comparing the recommendations of the Royal College of Psychiatrists with clinical practice^9,10^ and looking at strategies to improve its organisation.^11^ Recommendations such as improving the availability of supervision,^12^ protected time^13^ and suitable cases have been made.^14,15^ Although there has been literature published on the assessment of psychotherapy competencies for psychiatric trainees,^16,17^ to the best of our knowledge, there is little published on the competency of psychiatric trainees to actually deliver CBT based on structured feedback from supervisors and the use of structured tools such as those found on postgraduate psychotherapy courses.

Cognitive–behavioural therapy supervision sessions are based on an established format.^12^ This incorporates agenda-setting, case discussion/review of session, didactic discussion of the CBT model for the presenting problem, questions for supervision, plan for next session with patient, homework task (e.g. recommended reading) and audio/videotape review if available. Trainees are expected to work with their patients for up to 12 sessions and are encouraged to record their sessions (audio or video) with the patient’s consent. Trainees should attend regular supervision sessions with their allocated supervisor. Excerpts from the therapy session recordings are listened to and used as a tool to guide the trainee’s supervision.

In addition to structured supervision reports, a further method of establishing trainee competence is the revised Cognitive Therapy Scale (CTS-R).^18,19^ The CTS-R is widely used in postgraduate CBT training courses as a way of grading course work and, although not without controversy, is considered the gold-standard measure of clinical competence.

We hypothesised that psychiatric trainees would perform effectively and competently as CBT therapists (based on structured supervision reports) if well supervised and if allocated suitable, uncomplicated cases.

Our aims were as follows:

- to retrospectively review all available supervision reports for psychiatric trainees rotating through a national psychiatric hospital to investigate their competency at delivering CBT;
- to investigate whether CTS-R reports, where available, supported the findings of the supervisor’s report;
- to investigate trainee satisfaction with receiving CBT supervision.

## Method

The study was undertaken in St Patrick’s University Hospital, a 300-bed facility in Dublin affiliated with the University of Dublin, Trinity College. It has a well-developed psychotherapy service. The hospital receives trainees from the Dublin University Psychiatric Regional Training Programme (DUPRTP) on 6-month rotations.

Since 2009, there has been a single, time-protected psychotherapy post on the DUPRTP located in this hospital, with supervision provided by a consultant psychiatrist with psychotherapy training. The time for this CBT delivery is protected by another trainee covering their work. Obtaining this post is a competitive process and trainees are expected to complete at least one case using CBT during their rotation.

All therapists working in the hospital have been accredited by the British Association of Behavioural and Cognitive Psychotherapies (BABCP) and are involved in training and examining on the cognitive psychotherapy course, University of Dublin, Trinity College, which uses the CTS-R extensively.

All trainees at the outset of their 6-month rotation in St Patrick’s University Hospital were invited to provide psychotherapy with CBT under supervision. Initial consultant-led teaching is provided on basic psychotherapy and CBT skills. This comprises of three introductory 2-hour seminars for each 6-month intake of trainees at the hospital. The teaching sessions comprised: introduction to the CBT model; cognitive distortions; structuring a session; use of behavioural techniques; guided discovery and Socratic questioning; planning a course of therapy; and use of supervision.

The cases undertaken by the trainees were recruited from both out-patient and in-patient CBT waiting lists and assessed for suitability prior to allocation. Suitable training cases were individuals deemed to have a typical Axis 1 disorder^20^ without active complications or comorbidities, who were easy to engage interpersonally. Trainees were encouraged to record their sessions using audiotape or videotape – with patient consent – for discussion at supervision. Supervision sessions occurred fortnightly.

Following the end of therapy, supervisors completed a structured report used by the hospital’s psychotherapy service for rating trainees, using the common headings: Establishing a therapeutic relationship; Ability to apply CBT model; Understanding of model preparation; Use of supervision time; and Overall. They rated trainees using a simple Likert scale (0–6) that was incorporated from the CTS-R^18^ and work by Dreyfus.^19^ Ratings are: 0, negative impact; 1, no impact (neutral); 2, minimal impact; 3, some positive impact; 4, moderately successful impact; 5, successful impact; and 6, highly successful impact. A result of 3 or over for each item indicates competence.

All trainees under supervision were invited to submit an audio/videotape recording to be assessed using the CTS-R. The CTS-R^18^ is a revised version of the existing Cognitive Therapy Scale.^21^ The rater assesses trainee competence in 12 areas:

1. agenda setting and adherence
2. feedback
3. collaboration
4. pacing efficient use of time
5. interpersonal effectiveness
6. eliciting appropriate emotional expression
7. eliciting key cognitions
8. eliciting behaviours
9. guided discovery
10. conceptual integration
11. application of cognitive change
12. homework setting.

Ratings are given using the same 7-point Likert scale as described above to establish the trainee’s competency in each area. A result between 36 and 48 from a total of 72 establishes competency in that assessment.^18,21^ The CTS-R has demonstrated high internal consistency and interrater reliabilty.^22^

Trainee satisfaction with the quality of supervision and free-text comments were also recorded using a specifically designed questionnaire (details available from the authors on request). Trainees were asked about: availability of supervision; atmosphere conducive to feedback; availability of suitable cases; supervisor’s ability to communicate theory; and an overall rating.

Following approval by the hospital’s ethics committee, we reviewed all available supervisor reports and CTS-R ratings made between July 2004 and December 2012.

## Results

**Fig. 1:**
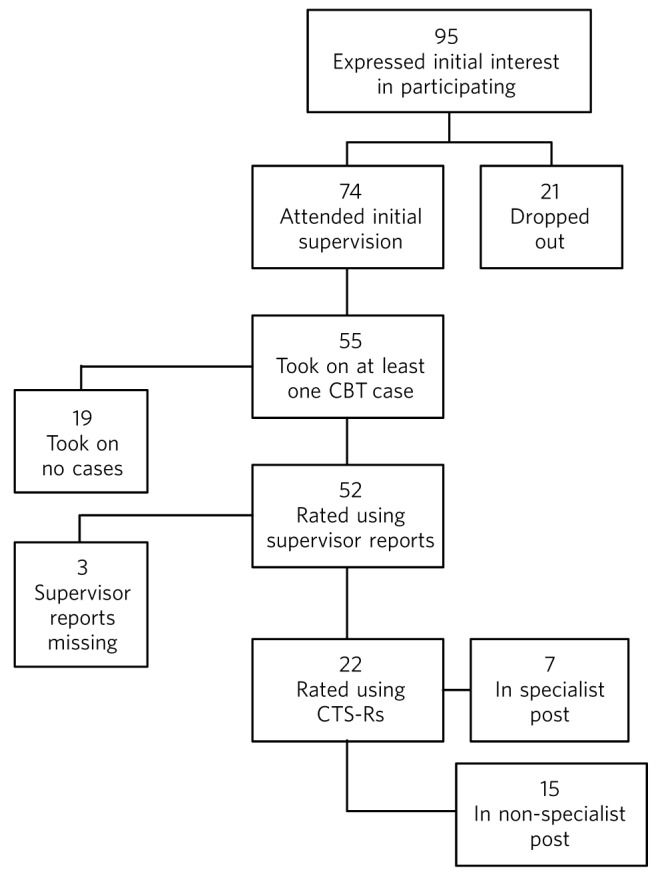
Flow chart showing the number of trainees in the study. CBT, cognitive-behavioural therapy; CTS-R, revised Cognitive Therapy Scale.

Over an 8.5-year period, 95 trainees expressed interest in participating in training to treat a patient using CBT (Fig. 1). Twenty-one trainees subsequently dropped out, citing lack of free time for psychotherapy and work pressures.

Seventy-four trainees attended introductory lectures and were allocated to a CBT supervisor. Of these, 37 attended supervision with nurse therapists and 37 attended supervision with the consultant. Nineteen trainees attended an initial supervision session but could not recruit a training case or a suitable training case was not available. Of the 95 trainees, 55 (58%) treated at least one patient using a CBT model.

Complete data were available for 52 of the 55 participants (95%). The remaining three supervisor reports were not completed or could not be located. Of the 55 participating trainees, 7 rotated through protected psychotherapy.

### Characteristics of trainees

In total, 55 trainees treated a patient using CBT (55% male, mean age 31 years (range 25.1–42.8)). All were psychiatric trainees pre-membership (MRCPsych) with no previous experience of delivering CBT. Trainees had spent a mean of 15 months (s.d. = 8.2) in psychiatric training.

Of the 55 trainees, 7 completed a protected training post. Characteristics of this subgroup were well matched to other trainees (43% male, mean age 31 (range 26.5–32.8)).

In total, 38 trainees took on 1 case, 14 trainees took on 2 cases, and 3 trainees took on 3 cases (total = 76 patients). Twenty-two trainees (40%) availed of the opportunity to have an assessment rated using the CTS-R. For those trainees who saw more than one case, we reviewed their CTS-R from their first case only.

### Patient characteristics

Of the 76 patients seen, the initial working diagnoses included depression (*n* = 21), obsessive-compulsive disorder (*n* = 12), social anxiety (*n* = 10), panic disorder (*n* = 7), generalised anxiety (*n* = 7), health anxiety (*n* = 3), low self-esteem (*n* = 2) specific phobia (*n* = 2), eating disorder (*n* = 2), non-epileptic seizures (*n* = 1), behavioural activation (*n* = 3), anger management (*n* = 3), psychosis (*n* = 2) and borderline personality (*n* = 1).

### Supervisors’ structured ratings of all trainees delivering CBT

**Fig. 2:**
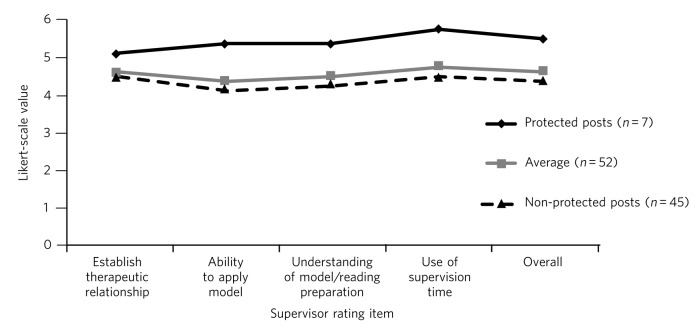
Comparison of structured ratings for trainees in protected and unprotected posts. Likert scale values are itemised as: 0, negative impact; 1, no impact (neutral); 2, minimal impact; 3, some positive impact; 4, moderately successful impact; 5, successful impact; 6, highly successful impact.

The average result for the complete data available for the 52 trainees was found to be at or above the accepted level for competency (≥3) across a range of areas. Results for each item were: Establishing a therapeutic relationship, mean = 4.6, s.d. = 0.7; Ability to apply model, mean = 4.4, s.d. = 0.9; Understanding of the model/reading preparation, mean = 4.5, s.d. = 0.9; Use of supervision time, mean = 4.7, s.d. = 0.9; and Overall, mean = 4.6, s.d. = 0.9. Trainees in the protected post (*n* = 7) scored higher in all areas of the supervisor’s report compared with non-protected posts (*n* = 45). The greatest difference was seen in trainees’ ability to apply the CBT model and the use of supervision time (Table 1 and Fig. 2).

There was a significant difference (*P*<0.001) in the supervisor scores obtained by those trainees (*n* = 22) who submitted an audio/videotape to be reviewed using the CTS-R (mean = 4.9, s.d. = 0.158) compared with the remaining (*n* = 30) trainees (mean = 4.28, s.d. = 0.13): *t*(50) = 15 (Table 2). Of the 22 trainees who submitted a tape, 7 were in the protected psychotherapy post. The remaining trainees (*n* = 15) were in a range of general adult psychiatry posts.

The average rating for all 22 trainees was 41.74 (s.d. = 5.16). Trainees were rated highest in interpersonal effectiveness (4.14) and eliciting key behaviours (3.89) and cognitions (3.63), and lowest at eliciting appropriate emotional expression (2.98). The results of the CTS-R findings are displayed in Table 3.

### Trainees’ ratings of supervisors

Of the 55 trainees, 49 (89%) rated their satisfaction with supervision over the training period. Six trainees did not return forms. The majority reported the supervision they received as either excellent, very good or good in separate areas: availability of supervision (48/49, 97%); atmosphere conducive to feedback (49/49, 100%); availability of cases (41/49, 84%); supervisor’s ability to communicate theory (49/49, 100%); and overall satisfaction (49/49, 100%). Remaining trainees rated the availability of cases as adequate or unsatisfactory and 1 trainee rated the availability of supervision as unsatisfactory.

Free-text section feedback suggested that trainees wished for more opportunities to continue with psychotherapy training, more suitable training cases to apply the CBT model and more protected time. Those who agreed to have an audio/videotape reviewed using the CTS-R found it helpful for guidance as to what areas to focus on for future therapy sessions. Anecdotal feedback from supervisors suggested that the training experience was positive; however uncontracted ‘goodwill’ supervision of psychiatric trainees was felt to place an unsustainable extra demand on busy CBT practitioners. Arranging CBT supervision within the same multidisciplinary team was deemed ideal as the psychiatric trainee can take on cases that would have been allocated to the team’s CBT practitioner.

**Table 1:** Comparison of mean structured ratings for trainees in protected and non-protected and non-protected posts

Supervisor rating	Protected posts (*n* = 7) mean score	Non-protected posts (*n* = 45), mean score	Overall (*n* = 52) mean score
Establish therapeutic relationship	5.1	4.5	4.6
			
Ability to apply model	5.4	4.2	4.4
			
Understanding of model/reading preparation	5.4	4.3	4.5
			
Use of supervision time	5.7	4.5	4.7
			
Overall	5.5	4.4	4.6

**Table 2:** Comparison of mean structured supervisor ratings for trainees who did (+) and did not (–) submit a recording to be rated using the revised Cognitive Therapy Scale (CTS-R)

Supervisor rating item	Mean supervisor rating CTS-R (+) (*n* = 22)	Mean supervisor rating CTS-R (–) (*n* = 30)	Overall mean (*n* = 52)
Establish therapeutic relationship	4.8	4.5	4.6
			
Ability to apply model	4.7	4.1	4.4
			
Understanding of model/reading preparation	4.8	4.2	4.5
			
Use of supervision time	5.1	4.3	4.7
			
Overall	5	4.3	4.6

## Discussion

We conducted a retrospective review of supervisor assessments for psychiatric trainees who, under supervision, engaged in a programme of delivering CBT to patients. The completion rate in our study (58%) was broadly similar to that reported in other similar studies^11^ evaluating a CBT training programme for psychiatric trainees. Our findings suggest that trainees can provide meaningful clinical interventions when delivering CBT under close supervision and with carefully selected cases. The mean rating for all trainees (i.e. 4.6, s.d. = 0.9) means that their CBT therapy had at least a moderately successful impact, which supports our primary hypothesis. Obstacles to participation cited by trainees included well-documented reasons of work pressures^11,15^ and lack of protected time.^13^ As the structured feedback shows, trainees who did participate found it a positive experience.

Trainees bring many strengths to the delivery of therapy as a result of their medical training, including knowledge of psychopathology and diagnostic systems and being used to working independently. Medicine as a profession has a strong academic base and doctors as professionals value characteristics such as ‘competence’.^23^ A particular strength for the trainees in this study was their ability to establish a therapeutic relationship with clients. Indeed, this ability forms the foundation for delivering

**Table 3:** Revised Cognitive Therapy Scale (CTS-R) ratings from 22 trainees who submitted a tape to be reviewed

CTS-R item	Mean rating (*n* = 22)
1. Agenda setting and adherence	3.14
	
2. Feedback	3.20
	
3. Collaboration	3.32
	
4. Pacing efficient use of time	3.93
	
5. Interpersonal effectiveness	4.14
	
6. Eliciting appropriate emotional expression	2.98
	
7. Eliciting key cognitions	3.63
	
8. Eliciting behaviours	3.89
	
9. Guided discovery	3.33
	
10. Conceptual integration	3.40
	
11. Application of cognitive change	3.58
	
12. Homework setting	3.20
	
Total (out of 72)	41.74 (s.d. = 5.16)

therapy itself.^24,25^ It is likely to have contributed to trainee success.

Trainees who were in a dedicated psychotherapy post (*n* = 7) obtained higher supervision scores than those who were not (*n* = 45). They were time-protected during their delivery of therapy and were immersed in a team environment dedicated to delivering CBT. Furthermore, in obtaining their psychotherapy post, they were self-selected as having already an established interest in delivering psychotherapy. These factors are likely to have contributed to them obtaining higher scores than those who did not have protected time.

The competency ratings using the CTS-R are in line with the structured supervisor reports. For those who were assessed using the CTS-R, trainees were rated highest in interpersonal effectiveness and eliciting key behaviours and cognitions. They rated lowest at eliciting appropriate emotional expression. This is in keeping with our experience of supervising psychiatric trainees. Eliciting emotional expression is challenging because it requires the trainee to leave their established role as a doctor and enter the more experiential role of a therapist.

There was a significant difference in supervisor ratings in favour of those trainees who submitted an audio/videotape to be reviewed using the CTS-R (*n* = 22) compared with those who did not. We propose that these self-selected trainees were inherently more confident at delivering CBT to patients, as they agreed to an additional rating scale using the CTS-R. Trainees who did not submit a tape for CTS-R review were not surveyed as to reasons why, which retrospectively would have been helpful. Encouraging and engaging more apprehensive trainees in psychotherapy training and specifically video feedback may be a challenge. In many ways it is these trainees who might benefit most from structured/objective feedback. Meeting this challenge requires a judicious blend of mandatory training requirements and a supportive, non-judgemental training environment.

This study has several limitations. To reliably assess competencies on the higher diploma in cognitive psychotherapy offered by Trinity College, for example, one would need to examine one case report, one essay, three tapes and a class presentation. This study employed structured supervisor reports completed at the end of therapy and CTS-R assessments in some cases. The CTS-R was assessed at one time point during the course of therapy and ideally two time points should have been used.^22^ Furthermore, although all supervisors had been accredited by the BABCP, ideally an external supervisor should have also assessed the CBT delivered by trainees to remove any bias.^11^ We do not have completed outcome measures from participating patients, which would have been useful.

With adequate planning, as in this study, trainees after a mean of 15 months’ training could treat selected cases with CBT, thus helping to address the demand for increased provision of ‘talking therapies’. Supervisors highlighted that some supervision was delivered on a ‘goodwill’ basis and suggested that the cases chosen should come from the list of that particular team’s allotted therapist, thus helping to reduce their workload and enable them to provide supervision and protect their time. Trainees themselves can support their competencies by using structured outcome measures and session recordings when providing CBT. This can further help to demonstrate the therapeutic value of trainees’ CBT casework in resource-pressured clinical services.

Training in psychotherapy such as CBT affords the trainee the opportunity to enrich their role as a psychiatrist and gain valuable skills that can help them and the patients they treat throughout their career. In addition, it provides trainees with a valuable insight into a therapeutic intervention that they will be either delivering themselves or referring to another provider. As indicated in this study, trainees can provide meaningful clinical interventions when delivering CBT under close supervision and with carefully selected cases. The costs of training and supervision need to be judged against these clinical gains.
